# Supplementary material for: miR3633a-GA3ox2 Module Conducts Grape Seed-Embryo Abortion in Response to Gibberellin
Source: Int J Mol Sci. 2022 Aug 7;23(15):8767. doi: 10.3390/ijms23158767 (PMC9369392; doi:10.3390/ijms23158767)
Supplement: Supplementary file 1 [file ijms-23-08767-s001.zip › Table S1.pdf]

Table S1 Basic information of gibberellin oxidase family gene

Type	Gene name	Gene number	Length	Chromosome location
GA3ox	*VvGA3ox-like*	VIT_09s0002g05270	1877	Chr9:4993413..4995290
	VvGA3ox2	VIT_09s0002g05300	3309	Chr9:5037569..5040878
GA20ox	*VvGA20ox1*	VIT_15s0048g01320	1597	Chr15:15453533..15455130
	VvGA20ox2	VIT_04s0044g01650	1767	Chr4:23382368..23384135
	VvGA20ox3	VIT_16s0022g02310	1795	Chr16:14861395..14863190
GA2ox	*VvGA2ox1*	VIT_19s0140g00140	1723	Chr19:15603207..15604930
	VvGA2ox2	VIT_05s0077g00520	2789	Chr5:343990..346779
	VvGA2ox3	VIT_19s0140g00120	1723	Chr19:15603207..15604930
	VvGA2ox4	VIT_01s0010g01650	1945	Chr1:17965019..17966964
	VvGA2ox6	VIT_19s0177g00030	6673	Chr19:5864698..5871371
	VvGA2ox7	VIT_10s0003g03490	1647	Chr10:5846741..5848388
	VvGA2ox8	VIT_10s0116g00410	5022	Chr10:190065..195087
