# Supplementary material for: miR3633a-GA3ox2 Module Conducts Grape Seed-Embryo Abortion in Response to Gibberellin
Source: Int J Mol Sci. 2022 Aug 7;23(15):8767. doi: 10.3390/ijms23158767 (PMC9369392; doi:10.3390/ijms23158767)
Supplement: Supplementary file 1 [file ijms-23-08767-s001.zip › Table S2.pdf]

Table S2 Basic information of oxidase protection enzyme gene

Type	Gene name	Gene number	Length/bp	Chromosome location
SOD	*VvMnSOD*	VIT_13s0067g02990	3868	Chr13:1602971..1606839
	VvCuSOD	VIT_06s0061g00750	3992	Chr6:18276926..18280918
	VvCn/ZnSOD	VIT_08s0007g07280	3544	Chr8:20888585..20892129
	VvFeSOD	VIT_10s0042g00100	6944	Chr10:12756583..12763527
	VvFeSOD3	VIT_16s0013g00260	5710	Chr16:5200379..5206089
CAT	*VvNADPH-E*	VIT_11s0016g00540	5775	Chr11:542212..547987
	VvNADPH-C	VIT_19s0014g02830	5113	Chr19:2924506..2929619
	VvFeRO2	VIT_15s0046g01900	3823	Chr15:18731174..18734997
	VvFeRO7	VIT_12s0035g02150	8437	Chr12:22531017..22539454
	VvFeRO8	VIT_17s0000g09160	3128	Chr17:10745402..10748530
