# Supplementary material for: miR3633a-GA3ox2 Module Conducts Grape Seed-Embryo Abortion in Response to Gibberellin
Source: Int J Mol Sci. 2022 Aug 7;23(15):8767. doi: 10.3390/ijms23158767 (PMC9369392; doi:10.3390/ijms23158767)
Supplement: Supplementary file 1 [file ijms-23-08767-s001.zip › Table S3.pdf]

Table S3 qRT-PCR primer sequence

Name	Forward primer sequence	Reverse primer sequence
VvMIR3633a	AAACTGGAAAGCTGGCATGG	TCCCATTTCAAACCTGCAGC
VvGA3ox2	TTTGCCAAGCAAATGTGGTA	TCTGGACCAGCCCATTCTAC
VvGA3ox-like	GGCAGGAGCCTTTTCTCTCT	GCCTCTCTGCAAGTTGGTTC
VvGA20ox1	CCTGCACAGAGCAGTGGTAA	TGAATTCAAGCAGCGATGAC
VvGA20ox2	GCCAGGCTTCTTCTTGACAC	AAGGTGATGAGGCTGCAACT
VvGA20ox3	AATTCGGGCAAGTGTACCAG	CAATGAGGCCCTGTTCCTAA
VvGA2ox1	ATTCCCGGTTATTCCTACGG	AAATGAGCTCCAGTGGGATG
VvGA2ox2	CTGTGTTAACGTGGGGGACT	GAGAAGGCCTCTCAGGTGTG
VvGA2ox3	TGCCAAGAGTTTGGCTTCTT	GAAGGTACTCAACCCGACCA
VvGA2ox4	CTGTCAGTGTGCGGGACTAA	AAAGGCCATGTCAAACCTTG
VvGA2ox6	CTCCACTTGCTCACGATGAA	CGACGTGGATGAATGTGAAC
VvGA2ox7	TATCGCAGAATGGTCGTCAG	CAGCCAGGTCCTTCTCAGAC
VvGA2ox8	GGTTGATGCCACACACTGAC	TCTGGGTTGGGTTTAACTGC
VvMnSOD	GCAGTCTTCCATGGCTCTTC	GCTGCTCTAGGGCTTTGTTG
VvCuSOD	GCACAGCACGTTGTGCTAAT	CATGCCAAGTGGCAACATAC
VvCn/ZnSOD	TGGCAGAGTTGGATGTGGTA	CGAGGATTGAAGCCCAATTA
VvFeSOD	GGGATCCTTTCAACCAGACA	CAACTCATCTGGGTGGAGGT
VvFeSOD3	TTTCACATGCATTTGGGCTA	ATGACCAAACCCAGTTGAGC
VvNADPH-E	TGTTGGCTGCCTCTCTTTTT	CCTCTACTTCCACCGCTGAG
VvNADPH-C	GAGAATGGGTGCCTGTTGAT	CTTCACATGCAAAAGCCTGA
VvFeRO2	TCCAGAGGACAGCTCCACTT	CACCAGCGTCAGCAATTCTA
VvFeRO7	GAGGTGCTGTGGTTGGTTTT	GGCCTGCAACCATACTGAAT
VvFeRO8	GACTTGCATCCTGTCAGCAA	GATGCTTGTTGGGGCATACT
VvActin	GCTCGCTGTTTTGCAGTTCTAC	AACATAGGTGAGGCCGCACTT
