# Supplementary material for: miR3633a-GA3ox2 Module Conducts Grape Seed-Embryo Abortion in Response to Gibberellin
Source: Int J Mol Sci. 2022 Aug 7;23(15):8767. doi: 10.3390/ijms23158767 (PMC9369392; doi:10.3390/ijms23158767)
Supplement: Supplementary file 1 [file ijms-23-08767-s001.zip › Table S4.pdf]

Table S4 Amplification primers

Type	Name	Forward primer sequence	Reverse primer sequence
Gene	*VvMIR3633a*	AAATTCTTGATTTTGAATGACTCG	TCACTTGCGTCATTCGTATTAGA
Gene	*VvGA3ox2*	ATGCCTTCAGAACTCTCTGATG	CTCCGATGAATCCGTTTCC
Gene	*mVvGA3ox2*	ATGCCTTCAGAACTCTCTGATG	TGGACCAGCCCATTCTAC
Promoter	*VvMIR3633a*	GGCTCGAAATCTTAAGTTACACTC	AATCTATGTTTGATCGGTTGG
Promoter	*VvGA3ox2*	GAGAGAACATTCAAGATTGCAT	GTTGTGTTTTGCATGGCTGTCG
